# LHH1, a novel antimicrobial peptide with anti-cancer cell activity identified from *Lactobacillus casei* HZ1

**DOI:** 10.1186/s13568-020-01139-8

**Published:** 2020-11-11

**Authors:** Jun-Fang He, Du-Xin Jin, Xue-Gang Luo, Tong-Cun Zhang

**Affiliations:** 1 Key Lab of Industrial Fermentation Microbiology of the Ministry of Education & Tianjin Key Lab of Industrial Microbiology, College of Biotechnology, Tianjin University of Science and Technology, Tianjin, 300457 P. R. China; 2 School of Food Science and Engineering, Yangzhou University, Yangzhou, 225127 Jiangsu China; 3 State Key Laboratory of Food Nutrition and Safety, Tianjin, 300457 P. R. China; 4 Tianjin Engineering Research Center of Microbial Metabolism and Fermentation Process Control, Tianjin, 300457 China

**Keywords:** Antimicrobial peptide, Anti-cancer, *Lactobacillus casei*, *Staphylococcus aureus*, Pathogenic bacteria

## Abstract

Antimicrobial peptides have been attracting increasing attention for their multiple beneficial effects. In present study, a novel AMP with a molecular weight of 1875.5 Da, was identified from the genome of *Lactobacillus casei* HZ1. The peptide, which was named as LHH1 was comprised of 16 amino acid residues, and its α-helix content was 95.34% when dissolved in 30 mM SDS. LHH1 exhibited a broad range of antimicrobial activities against Gram-positive bacteria and fungus. It could effectively inhibit *Staphylococcus aureus* with a minimum inhibitory concentration of 3.5 μM and showed a low hemolytic activity. The scanning electron microscope, confocal laser scanning microscope and flow cytometry results showed that LHH1 exerted its antibacterial activity by damaging the cell membrane of *Staphylococcus aureus*. Meanwhile, LHH1 also exhibited anti-cancer cell activities against several cancer cells via breaking the cell membrane of MGC803, HCT116 and C666-1 cancer cells.

## Introduction

Over the last decades, human health was seriously threatened by pathogenic bacteria-induced diseases. Antimicrobial drugs and chemical preservatives such as antibiotics, sulphonamides and 4-quinolones have been commonly used to protect human from the infections of pathogens. However, the overuse of antimicrobial drugs has led to drug-resistance in many strains of pathogenic bacteria and efficiency loss to inhibit pathogenic microbes (Alekshun and Levy 2007). The pathogenic bacteria causing infections tend to adapt to new environments and hosts, and readily develop resistance to the anti-infective antibiotics used to treat them (Heymann 2006). The World Health Organization has described a worldwide resistance problem in bacterial species such as *Escherichia coli*, *Klebsiella pneumoniae* (*K. pneumoniae*), *Staphylococcus aureus* (*S. aureus*) and *Streptococcus pneumoniae* (Organization 2012). For example, with widespread use of penicillin, the prevalence of penicillin-resistant hospital strains of *Staphylococcus* had risen to no less than 95% by the end of the 1990s, and *Staphylococcus aureus* had accumulated resistance genes to virtually all currently available antimicrobial drugs, with methicillin-resistant infections disseminated in hospital settings and communities (Davies and Davies 2010). Nevertheless, antibiotics and antibiotic resistance mechanisms have appeared by natural selection and have a long evolutionary history, and most of the antibiotics have specific targets (Hincapie et al. 2018). Therefore, novel antimicrobial drugs will be required to treat the resistant species.

Antimicrobial peptides (AMPs), which are generally comprised of 10–60 amino acid residues, have been commonly regarded as promising antibiotic alternatives with a broad-spectrum antimicrobial activity (Cheung and Otto 2018). As an evolutionarily ancient weapon to innate immunity, AMPs can protect human from invading pathogens such as bacteria, fungi, viruses and parasites (Mishra et al. 2017). Moreover, AMPs have been found in various species including multicellular plants, animals and microorganism (Aminov 2017). For example, LL-37 was isolated from a human bone marrow library (A 1996), Leucocin A was a small heat-stable bacteriocin produced by Leuconostoc gelidum UAL187 (Belkum 1995), γ-Purothionins was extracted from wheat endosperm (Colilla et al. 1990).

Previous studies have reported some cationic AMPs possess anticancer activities (Bhattacharjya et al. 2015). For example, BmKn2 as an AMP showed inhibitory effect on colon carcinoma cells (Arpornsuwan et al. 2014), LL-37 and it’s analogs exhibited anticancer effects on several cancer cell lines (Kuroda et al. 2015), human intestinal defensin 5 could suppress the growth of colon cancer cells induced by 1,2-dimethylhydrazine dihydrochloride (Panjeta and Preet 2020).

Lactic acid bacteria are probiotics that have been recognized as safe with a broad application in food and pharmaceutical industries. Moreover, they possess the capability of producing AMPs (Pangsomboon et al. 2009).Nisin was the first bacteriocin identified from *Lactococcus lactis*, which has been acknowledged as safe by Food and Drug Administration and approved for application in food industry (Cotter 2005). Wen et al. reported that plantaricin K25 produced by *Lactobacillus plantarum* showed antibacterial activity against Gram-positive and Gram-negative bacteria (Wen et al. 2016). Meanwhile, pediocin and lacticin that produced by several species of *Pediococcus* and *Lactococcus* have been permitted to use in food product (Kaya and Simsek 2019). Moreover, several AMPs such as Nisin Z and Plantaricin A from lactic acid bacteria exhibited anticancer activity (Mulders et al. 1991; Nissen-Meyer et al. 1993). *Lactobacillus casei* (*L. casei*) HZ1 is also a species in lactic acid bacteria that was isolated from Chinese traditional fermented milk. Our previous study found a novel AMP (LGH2) from the genome of *L. casei* HZ1, which showed an excellent inhibitory activity against Gram-positive bacteria (He et al. 2018). However, to our knowledge, only a few studies have been conducted on the antimicrobial and anticancer activities of AMPs from *L. casei*. Identification of more AMPs can provide structure templates for the production of antimicrobial and anticancer drugs. Hence, further research on AMPs from the genome of *L. casei* HZ1 is essential.

In the present study, we attempted to detect some novel AMPs with anti-cancer cell activities from the genome of *L. casei* HZ1, and to explore the functional mechanisms of the AMPs against pathogenic bacteria and cancer cells. The hemolytic activity was also characterized for cytotoxicity against red blood cells.

## Materials and methods

### Materials

The AMPs and FITC-labeled AMPs were chemically synthesized by Bootech Bioscience & Technology Co., Ltd. (Shanghai, China). The synthesized peptides were purified by reversed-phase high-performance liquid chromatography (RP-HPLC) and were verified by mass spectrometry. The purities of both labeled and unlabeled peptides were above 98%. Melittin with purity of above 97% was purchased from the Sigma-Aldrich (Shanghai, China) (Additional file 1).

### Identification of potential AMPs from the genome of L. casei HZ1

The prediction of AMPs from the genome of *L. casei* HZ1 was conducted according to our previous research with some modifications (He et al. 2018). Briefly, the prediction of antimicrobial peptides conforms to the following principles: (1) An AMP should contain a Gly-Gly/Ala leader motif at the front of N-terminal region of mature AMPs. (2) The peptide might form α-helix structure. (3) The peptide is positive-charged. (4) Protein-binding potential should be above 0 kcal/mol. The APD3 (https://aps.unmc.edu/AP/prediction/prediction_main.php) was used to calculate the net charge, protein-binding potential and hydrophobic amino acid content in the peptide. The CAMP_R3_ (https://www.camp.bicnirrh.res.in/prediction.php) was used to calculate the antibacterial capacity of the peptides. The molecular weight was calculated by PeptideMass (https://web.expasy.org/peptide_mass/). The secondary structure model was constructed by PSIPRED (https://bioinf.cs.ucl.ac.uk/psipred/). The hemolytic property of peptides was predicted by HemoPI (https://crdd.osdd.net/raghava/hemopi/design.php) (Chaudhary et al. 2016). The genome of *L. casei* HZ1 was presented in the supplementary material.

### Antimicrobial activity

The antimicrobial activity of AMPs was measured by inhibition zone assay. Briefly, 200 μL of pathogenic bacteria (5 × 10^6^ CFU/mL) was added into 20 mL of Luria–Bertani (LB) solid medium and was spreaded evenly. Then, the round filter papers dampened with the AMPs were placed on the solid medium and were cultured at 37 °C for 18 h before recording the inhibition zones.

The minimun inhibitory concentration (MIC) was determined as described by Morita et al. with minor modifications (Morita et al. 2013). Briefly, the bacteria with a density of 1 × 10^6^ cells/mL was added into a 96-well plate. Then, AMP solution was added into each well to reach a final concentration of 0 to 256 μM, and the plate was incubated at 37 °C for 24 h. The MIC value was determined by OD600 readings in a microplate reader (Infinite M200 PRO, TECAN, Switzerland).

For the assessment of the maximum kill concentration (MBC), 5 µL of each well culture was added into another 96 well plate which was filled with 95 µL of fresh nutrient broth in each well and was incubated at 37 °C for another 24 h. The absorbance was determined at 600 nm. In order to get a more accurate data, extra points were added in the assay.

### Hemolytic activity

The hemolytic activity was determined with goat red blood cells according to the method described by Taute et al. (2015). Briefly, red blood cells were collected by centrifugation at 1000×*g* for 10 min before washing thrice with 50 mM Tris–HCl and were resuspended with 50 mM Tris–HCl containing 100 mM NaCl to reach a final concentration of 4% (V/V). 200 μL of the goat red blood cells were mixed with AMPs at different concentrations at 37 °C for 1 h. Then, the supernatant was collected by centrifugation at 1000×*g* for 10 min, and 100 μL of the supernatant was transferred into a 96-well plate. The absorbance was monitored at 550 nm. PBS and 0.1% Triton X-100 solution were used instead of AMP as negative and positive control, respectively.

1 $${\text{Hemolysis}}\;{\text{rate}} \left( \% \right) = \frac{{{A_{peptide}} - {A_{PBS}}}}{{{A_{Triton}} - {A_{PBS}}}} \times 100$$

where *A*_*peptide*_ is the absorbance of peptide treatment group, *A*_*PBS*_ is the absorbance of reaction in absence of peptides, and *A*_*Triton*_ is the absorbance of reaction treated with 1% Triton X-100 instead of peptide.

### Anticancer activity

HCT116 cell was cultured in 1640 medium with 10% (v/v) inactivated fetal bovine serum, while MGC803 and C666-1 cells were maintained in DMEM high glucose medium with 10% (v/v) inactivated fetal bovine serum. All cells were cultured in an incubator containing 5% CO_2_ at 37 °C and were harvested at 80% confluency. The cancer cells were plated at a density of 5 × 10^3^ cells/well in 96-well culture dishes and were incubated with AMPs of different concentrations for 24 h. Moreover, melittin with the same concentration as AMPs was added instead of the AMPs as a positive control. Then, 10 μL of MTT (5 mg/mL in PBS) was added into each well and was co-incubated at 37 °C for another 4 h. Subsequently, the precipitate was dissolved with DMSO after removing the medium, and the optical density was measured at 490 nm with a microplate reader. The toxicity effect of AMPs on RAW264.7 cells was detected to determine the cytotoxic effect of AMPs on normal cells. Moreover, melittin was added instead of LHH1 as a positive control in anticancer cell assay.

2 $${\text{Cell}}\;{\text{viability}}(\% ) = \frac{{A_{{control}} - A_{{peptide}} }}{{A_{{control}} }} \times 100$$

where *A*_*peptide*_ is the absorbance of peptide treatment group, and *A*_*control*_ is the absorbance of reaction in absence of peptides.

### Circular dichroismspectroscopy

The secondary structures of the peptides in different solutions were determined by a MOS-450 circular dichroism (CD) spectrometer (Bio-Logic, France) with a 0.5 cm quartz cell at 190–240 nm. The AMPs were dissolved in six solvents including water, sodium phosphate buffer (10 mM, pH 7.4), SDS micelles (30 mM), 25% TFE, 50% TFE and 500 mM phospholipid to a final concentration of 100 μM, respectively. An average of three scans was recorded for each sample. The acquired CD signal spectra was converted to the mean residue ellipticity. The secondary structures of the AMPs were calculated using the CD data at Dichroweb platform (https://dichroweb.cryst.bbk.ac.uk/html/links.shtml) using three different algorithms (SELCON3, CONTIN-LL and CDSSTR) (Whitmore and Wallace 2004).

### Scanning electron microscopy

The morphology of *S. aureus* was observed by scanning electron microscope (SEM). *S. aureus* was harvested at mid-logarithmic phase by centrifugation at 1000 × *g* at 4 °C for 10 min. The cells were diluted to a concentration of 1 × 10^8^ CFU/mL with 10 mM PBS (pH 7.4). Then, *S. aureus* dilution was incubated with the AMPs for 30 min. After washing the bacteria three times with 10 mM PBS at 1000 × *g* for 10 min, the cells were resuspended in 2.5% glutaraldehyde for 30 min and were washed thrice with PBS. Then, the cells were dehydrated by 10%, 30%, 50%, 70%, 80%, 90% and 100% ethanol, respectively. The dehydrated cells were dried by CO_2_ under critical point, and were sprinkled, gold-plated and imaged by SEM.

### Flow cytometry

The effect of AMPs on cell membrane integrity of *S. aureus* was determined by flow cytometry according to the method described by Xu et al. (2015). Briefly, *S. aureus* at mid-logarithmic phase was harvested by centrifugation at 1000×*g* for 10 min, washed thrice with PBS and re-suspended the bacteria with PBS to a density of 1 × 10^6^ cells/mL. Then, 300 μL of bacterial suspensions was mixed with 300 μL of AMPs to a final concentration of 10 μM. The mixture was incubated at 37 °C for 1 h followed by dyeing with 20 μM propidium iodide (PI) in dark at 4 °C for 15 min. For control, PBS instead of the AMP was added. The mixture was detected by flow cytometer (C6, BD, USA).

The cancer cells were plated into 6-well culture plates (1 × 10^6^ cells/well) and were treated with various concentrations of the AMP (5, 10, and 20 μM) or untreated. After incubation for 1 h, the cells were harvested by tryptic digestion, washed thrice with cold PBS, and resuspended with 1 × binding buffer. 1 × 10^5^ cells were then transferred to a 1.5 mL tube, and then 5 µL of FITC-conjugated Annexin V (BD Bioscience, USA) and 5 µL of propidium iodide (PI) were added. The cells were gently mixed, and incubated at room temperature in dark for 15 min. After incubation, 400 µL of 1 × binding buffer was added to each tube, the stained cells were measured by flow cytometre.

### Calcein leakage assay

The calcein-entrapped large unilamellar vesicle was prepared as described by Pu and Tang ( 2017). Briefly, 1-palmitoyl-2-oleoyl-sn-glycero-3-phosphoglycerol (POPG) and cardiolipin (CL) lipids (the ratio of POPG and CL was 58:42) were dissolved in chloroform, dried by nitrogen gas and were resuspended in dye buffer solution (10 mM HEPES, 50 mM calcein, pH 7.4). The suspension was subjected to 10 frozen-thaw cycles in liquid nitrogen and was extruded more than twenty times through polycarbonate filters with two stacked 100 nm pore size filters. Untrapped calcein was removed by Sephadex G-25 column. Finally, Calcein-loaded large unilamellar vesicles (LUVs) total phospholipid concentration was adjusted to a final concentration of 25 μM. The leakage of calcein was detected by measuring the fluorescence intensity at an excitation wavelength of 490 nm and an emission wavelength of 515 nm in a microplate reader. The 100% dye leakage was determined by 1% Triton X-100. The percentage of dye leakage was calculated by the equation as follows.

3 $${\text{Dye}}\;{\text{leakage}}\left( \% \right) = \frac{{F - {F_0}}}{{{F_{100}} - {F_0}}} \times 100$$

where *F*_*0*_ is the fluorescence intensity of liposomes without AMPs, *F* is the fluorescence intensity of liposomes treated with AMPs, *F*_*100*_ is the fluorescence intensity of liposomes treated with 1% Triton X-100.

### Confocal laser scanning microscopy

*S. aureus* at mid-logarithmic phase was diluted to a concentration of 10^6^ CFU/mL with PBS (pH7.4) and was co-incubated with labeled with fluorescein (FITC) synthetic peptide to reach a final concentration of 100 μM for 1 h. Then, the cells were washed thrice with PBS and were visualized with a confocal laser scanning microscope (CLSM) equipped with a Zeiss Neofluor 40× objective (numerical aperture 0.75).

For cancer cells, 1 × 10^6^ cancer cells were incubated with the optimal medium at 37 °C for 24 h. 10 mM FITC-labeled AMP was added and was co-cultured with the cells for 30 min before washing with PBS. Then, the cells were dyed with DAPI for another 10 min and were washed thrice with PBS. The cancer cells were observed using a CLSM at 40× objective lens.

### Statistical analysis

All experiments were carried out in thrice and the results were presented as the means ± standard deviation (SD) The data were analyzed with SPSS 19.0 and the level of significance were analyzed by one way analysis of variance (ANOVA). *P* < 0.05 were considered statistically significant.

## Results

### Prediction of AMPs from the genome of L. casei HZ1

To predict novel AMPs, the open reading frames (ORF) less than 450 bps in the genome of *L. casei* HZ1 were analyzed. Four novel potential AMPs were obtained and were blasted by NCBI and APD3 data bank, which showed that these four peptides had not been reported. The characteristics of the four AMPs were listed in Table 1. The four putative AMPs displayed a low hemolytic score, which indicated that they were low toxic. However, hydrophobic rate and helix contents of the four AMPs were obviously different. Compared with other three peptides, LHH1 contained a relatively higher content of hydrophobic amino acids and helix with a HR value and helix content of 62% and 81.25%, respectively. Moreover, LHH1 possessed the highest pI value of 11.71 and the lowest molecular weight of 1875.25 Da compared to the other three peptides. As shown in Table 2, both LHH1 and LHH4 shared the same motif of Gly-Gly at the N-terminus of the predicted AMPs, while LHH2 and LHH3 had the same motif of Gly-Ala at the N-terminus of the predicted AMPs.

**Table 1:** The characteristics of the four peptides

Name	Amino acid sequence	pI	NC	NA	HR	Helix	HS	MW
LHH1	AFALIAGALYRIFHRR	11.70	+ 3	16	62	81.25	0.49	1875.25
LHH2	EKAPEAYVKKIASLYRNKRY	9.87	+ 4	20	30	75.00	0.49	2427.82
LHH3	LIQFLEDNKKTTPHANAK	8.51	+ 1	18	33	31.25	0.51	2068.36
LHH4	FGVIVGHCLGHSGNWRKWIE	8.24	+ 1	20	45	15.00	0.48	2295.65

Net charge (NC) represents the total charge of an AMP; Number of amino acid residues (NA) is the total number of the peptide; Hydrophobic rate (HR) is the rate of hydrophobicity amino acid in the sequence of AMP, %; Helix is the content of helix structure in the secondary structure of AMPs, %. Hemolytic score (HS) indicates the hemolytic potency

**Table 2:** The location of the four peptides precursor in *L.casei* HZ1 genome

Name	Peptide precursor	Location in *L. case* HZ1
LHH1	(Aa)_50_ GG AFALIAGALYRIFHRR	LSEI_2135
LHH2	(Aa)_38_ GA EKAPEAYVKKIASLYRNKRY	LSEI_1186
LHH3	(Aa)_28_ GA LIQFLEDNKKTTPHANAK	LSEI_1507
LHH4	(Aa)_8_ GG FGVIVGHCLGHSGNWRKWIE	LSEI_0501

### Antimicrobial activity of the four putative AMPs

**Fig. 1:**
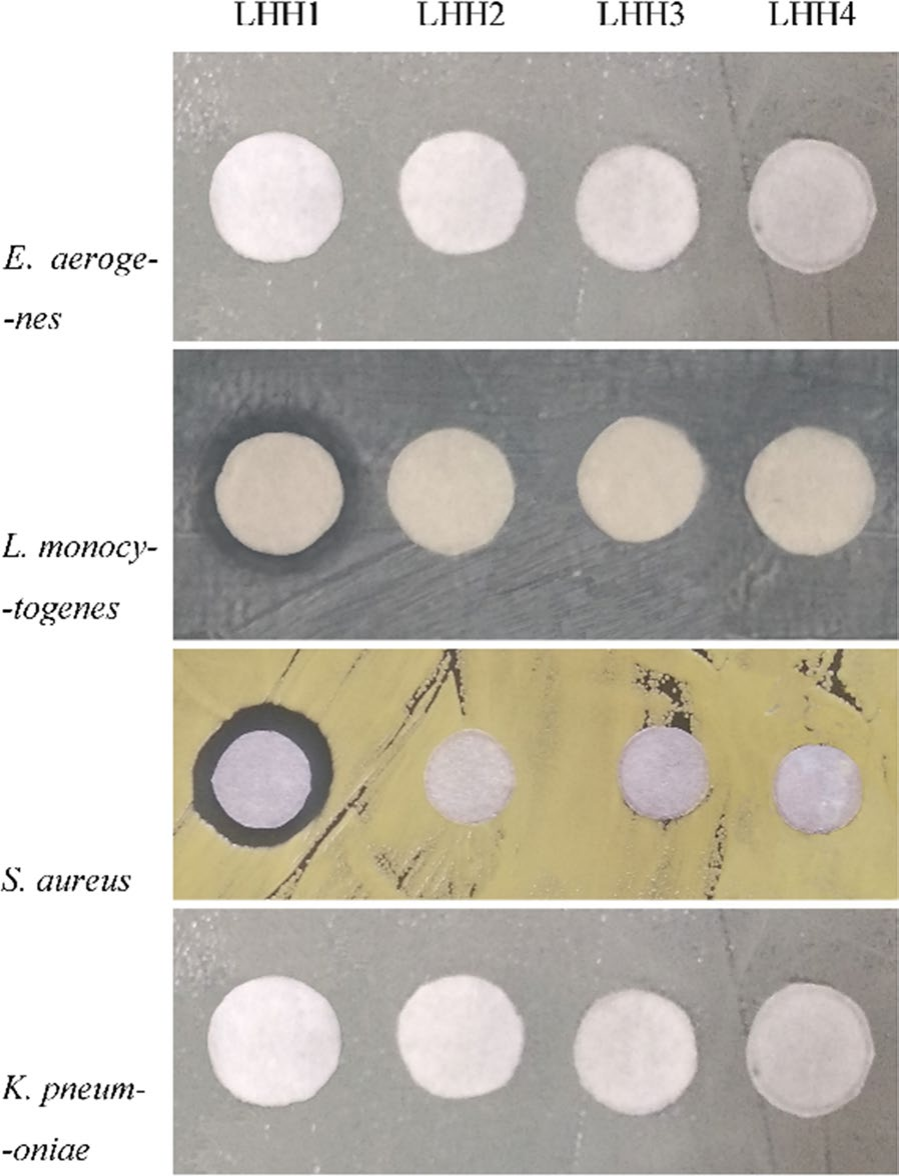
Inhibition zones of the four AMPs against several pathogenic bacteria

To test antimicrobial activities of the four predicted AMPs, an inhibition zone assay was applied to analyze the antimicrobial activities against several bacterial strains including *S. aureus*, *L. monocytogenes*, *K. pneumoniae* and *E. aerogenes*. Compared to the other three peptides, LHH1 showed an obvious inhibition zone against *S. aureus* and *L. monocytogenes* (Fig. 1). However, the other three AMPs showed no inhibitory effect against the tested pathogenic bacteria.

To further explore the antimicrobial activity of LHH1, inhibitory effect of LHH1 on eight strains including two fungus, four Gram-positive bacteria and two Gram-negative bacteria were evaluated in this study (Table 3). The results showed that LHH1 exhibited better inhibitory effect on several bacterial strains involving *S. aureus*, *M. luteus*, *L. monocytogenes* and *P. pastoris* GS115, all of which belonged to Gram-positive bacteria and fungus. The present results displayed that LHH1 possessed the strongest antimicrobial activity against Gram-positive bacteria, especially against *S. aureus* with a MIC value of 3.5 μM. Meanwhile, LHH1 exerted a weak inhibition on *L. casei*, which is a probiotics that beneficial to human health.

**Table 3:** Antimicrobial activity of LHH1

Microbial strains	LHH1		Ampicillin		Melittin	
	MIC (μM)	MBC (μM)	MIC (μM)	MBC (μM)	MIC (μM)	MBC (μM)
Gram−						
* Enterobacter aerogenes* (ATCC 13048)	> 256	> 256	8.00	16.00	8	16
* Klebsiella pneumoniae* (ATCC 700603)	> 256	> 256	32.00	64.00	16	32
Gram+						
* Staphylococcus aureus* (ATCC 29213)	3.50	6.50	0.25	0.50	4	8
* Micrococcus luteus* (ATCC 14452)	7.50	14.00	0.125	0.25	4	8
* Listeria monocytogenes* (ATCC 7611)	8.00	15.50	0.125	0.25	8	16
* Lactobacillus casei* (ATCC334)	> 256	> 256	0.5	1.0	4	8
Fungus						
* Saccharomyces cerevisiae* (ATCC 204508)	32.00	64.00	–	–	8	16
* Pichia pastoris* GS115 (ATCC 20864)	16.00	32.00	–	–	8	16

### Hemolytic activity of LHH1

**Fig. 2:**
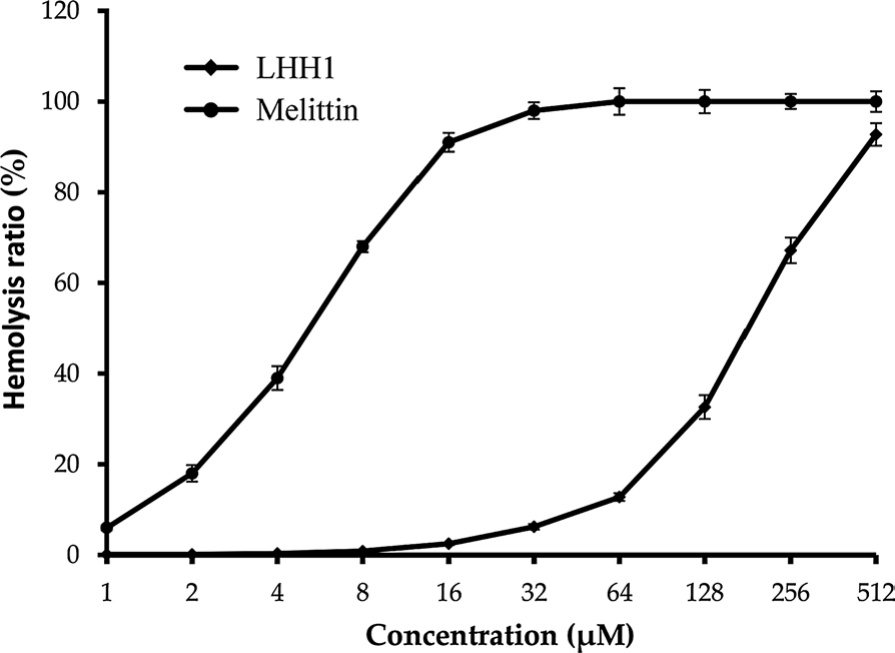
Hemolytic activity of LHH1. The goat red blood cell was treated by a serial concentration of LHH1 or Melittin from 1 µM to 512 µM

To confirm whether LHH1 was safe or not, its hemolytic activity was assessed. As showed in Fig. 2, LHH1 showed a low hemolytic activity against goat red blood cell when its concentration was lower than 64 μM. However, an obvious hemolysis was observed when its concentration was higher than 128 μM. The HC_50_ (hemolytic rate of 50%) value of LHH1 was 187.2 μM. Meanwhile, melittin exhibited an obviously high hemolysis with a HC50 value of 8.25 μM. The hemolysis results indicated that LHH1 exhibited an excellent inhibitory action on several Gram-positive bacteria at a low hemolytic concentration.

### Dye leakage assays

**Fig. 3:**
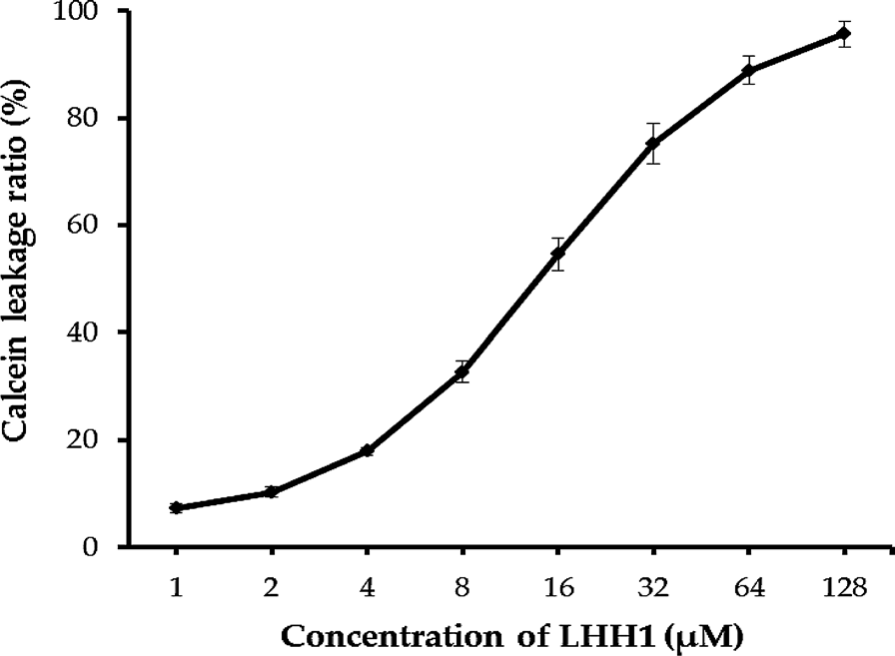
Release of calcein from liposomes. The concentration of LHH1 was from 1 μM to 128 μM

Calcein leakage assay is a common method to identify whether AMPs could interact with the phospholipids or not (Domingues et al. 2015). POPG and CL were two major cell membrane phospholipids of *S. aureus*, while calcein can be entrapped in LUVs composed of POPG/CL (58:42) (Epand 2008; Lohner and Prenner 1999). If AMPs could interact with the phospholipids, the LUVs would be damaged before the leakage of calcein. As shown in Fig. 3, calcein leakage ratio increased at a dose-dependent manner. LHH1 induced a calcein leakage content of over 50% at a concentration of higher than 15 μM. The calcein leakage results indicated that LHH1 could lead to the damage of phospholipid membrane of *S. aureus*.

### Secondary structure analysis of LHH1

**Fig. 4:**
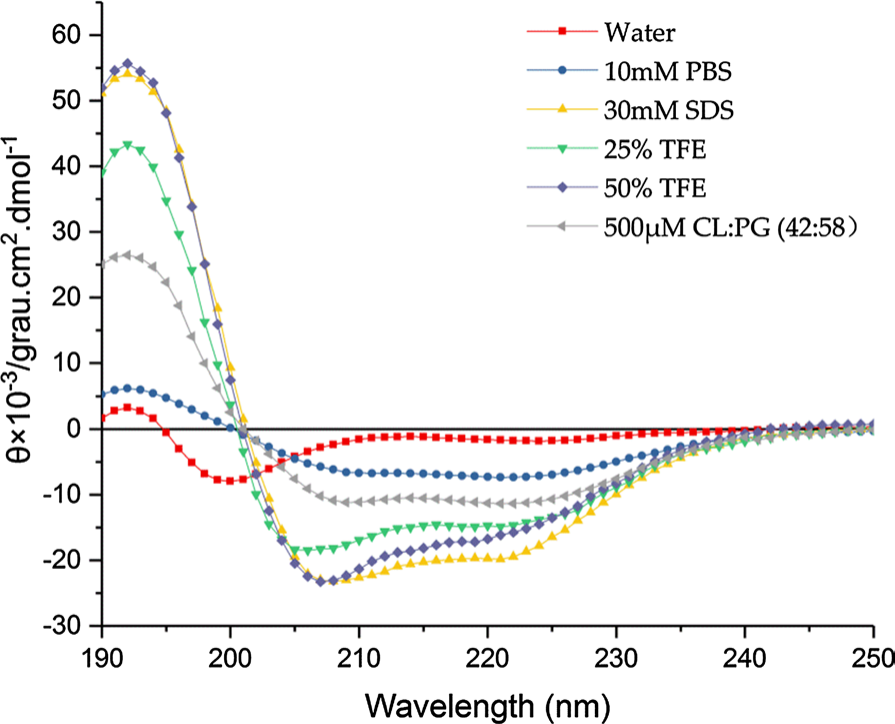
CD spectrum of LHH1. LHH1 was dissolved in water, 10 mM PBS (pH 7.4), 30 mM SDS, 25% TFE, 50% TFE and 500 μM POPG:CL (58:42) to reach a final concentration of 100 μM, respectively. Three scans were averaged for each sample

CD spectra has been considered as a powerful and sophisticated technique to analyze the secondary structure of protein (Hollosi 1994). Secondary structure of LHH1 dissolved in six different solutions was detected by CD spectra, and the results were shown in Fig. 4 and Table 4. LHH1 showed two negative peaks at 208 nm and 220 nm and a positive peak at 192 nm respectively when it was dissolved in 30 mM SDS, 25% TFE, 50% TFE and 500 μM (POPG:CL), which were the typical characteristics of α-helical structure in the peptides. However, LHH1 showed a relatively flat curve when it was dissolved in H_2_O and PBS. The contents of secondary structures of LHH1 in different solvents were determined by SELCON3 program (Whitmore and Wallace 2008). As shown in Table 4, LHH1 showed a high α-helix content of 95.29% and 95.34% when it was dissolved in 50% TFE and 30 mM SDS, respectively. Meanwhile, α-helical contents of LHH1 in 500 μM POPG:CL (58:42) was 73.05%. However, LHH1 mainly presented a structure of random coil when it was dissolved in water.

**Table 4:** Contents of secondary structures of LHH1 in different solvents

Solvent	Alpha-helix (%)	Beta-sheet (%)	Random coil (%)
Water	16.70 ± 2.50	4.85 ± 1.70	84.35 ± 3.10
PBS	47.48 ± 3.80	1.73 ± 0.70	52.57 ± 2.90
SDS	95.34 ± 3.10	0.02 ± 0.00	19.64 ± 1.50
TFE 25%	87.32 ± 3.70	0.04 ± 0.00	12.91 ± 0.00
TFE 50%	95.29 ± 3.0	0.02 ± 0.00	5.50 ± 0.40
500 μM (CL:POPG)	73.05 ± 2.80	0.26 ± 0.04	26.69 ± 0.60

### Membrane damage induced by LHH1

**Fig. 5:**
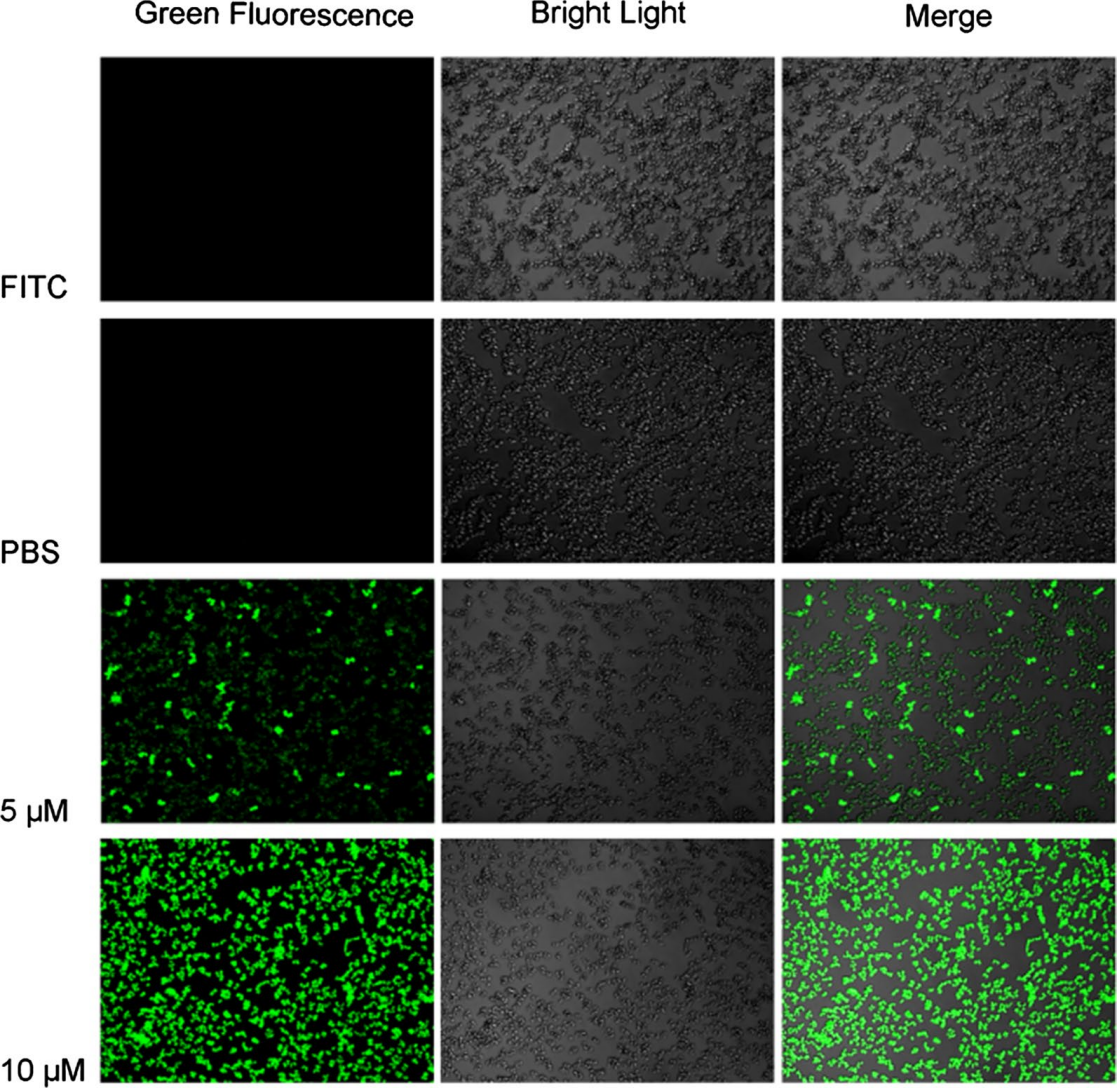
CLSM observation of *S. aureus* treated with LHH1. *S. aureus* was co-incubated with 10 μM FITC and 0, 5 μM or 10 μM FITC-labeled LHH1 for 1 h, and was observed with CLSM respectively

To explore whether LHH1 could bind to *S. aureus* or not, antibacterial mechanism of LHH1 against *S. aureus* was further studied by CLSM. As showed in Fig. 5, the fluorescence signal was clearly observed on the surface of *S. aureus* when it was treated with FITC-labeled LHH1, which indicated that LHH1 could bind to *S. aureus*.

**Fig. 6:**
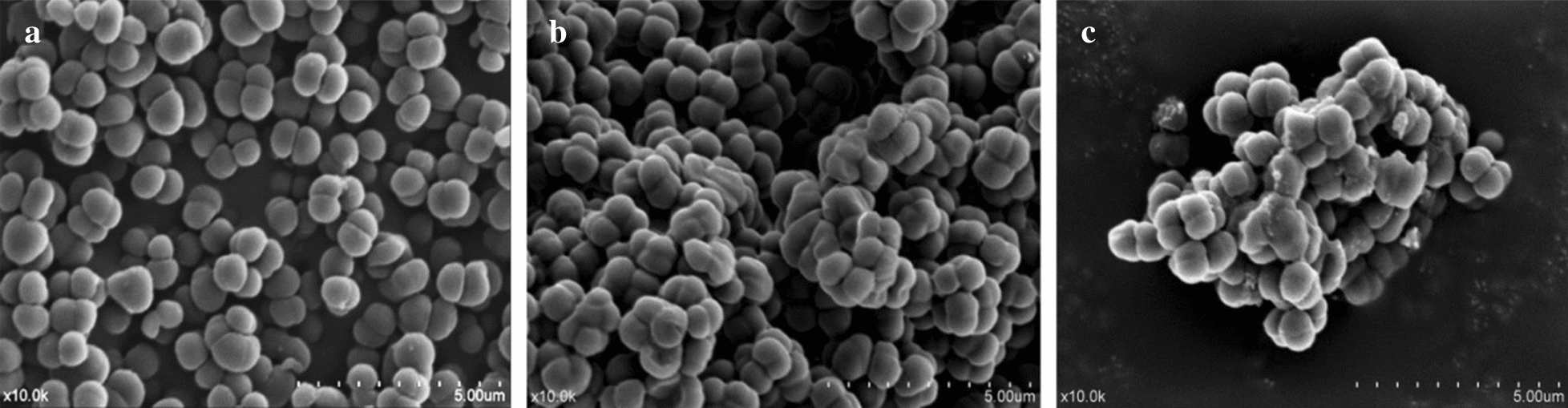
Morphology of *S. aureus* treated with different concentrations of LHH1. **a** *S. aureus* was not treated with LHH1; **b** *S. aureus* treated with LHH1 in 0.5 × MIC; **c** *S. aureus* treated with LHH1 in 0.75 × MIC; **d** *S. aureus* treated with LHH1 in 1.0 × MIC

To further confirm whether LHH1 could induce the membrane damage of *S. aureus*, SEM was utilized to observe the morphology of bacterial cells. As showed in Fig. 6, *S. aureus* showed a smooth and intact surface (Fig. 6a). However, LHH1 induced a roughened and deformed changes on the membrane of *S. aureus* at a dose-dependent manner. Meanwhile, *S. aureus* was covered with blebs, and some deformed and collapsed cells were observed when it was exposed to LHH1 of 0.5 × MIC or 1.0 × MIC (Fig. 6b, c).

**Fig. 7:**
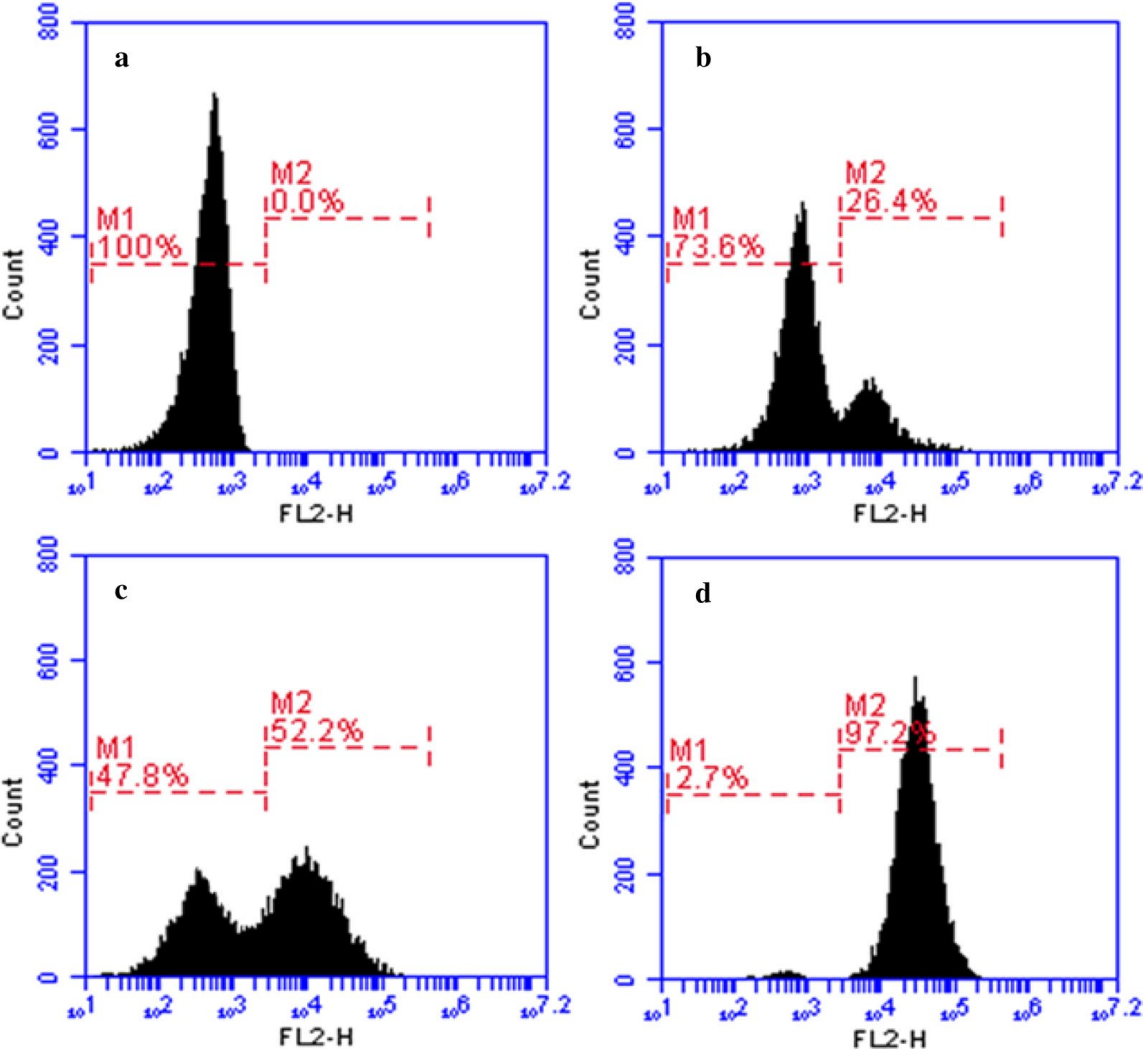
Flow cytometric analysis of the membrane integrity of *S. aureus. S. aureus* was treated with LHH1 at different concentrations at 4 °C for 30 min. **a** *S. aureus* of control group was treated with 10 mM PBS; **b** *S. aureus* was treated with LHH1 of 0.5 × MIC; **c** *S. aureus* was treated with LHH1 of 1.0 × MIC; **d** *S. aureus* was treated with LHH1 of 2.0 × MIC

To further explore the effect of LHH1 on membrane integrity of *S. aureus*, DNA intercalating PI was applied in this study. PI could penetrate into the cell and stain the nucleic acids if the membrane was damaged. As shown in Fig. 7, *S. aureus* showed no fluorescence when it was not treated with LHH1. However, the cells displayed a PI fluorescent signal after the treatment of LHH1, and the fluorescence intensity was positively correlated with the concentration of LHH1. LHH1 of 2 × MIC could cause approximately 97.2% cells of *S. aureus* stained by PI. Flow cytometry results indicated that LHH1 could induce the membrane damage of *S. aureus*.

### Anticancer cell activities of LHH1

**Fig. 8:**
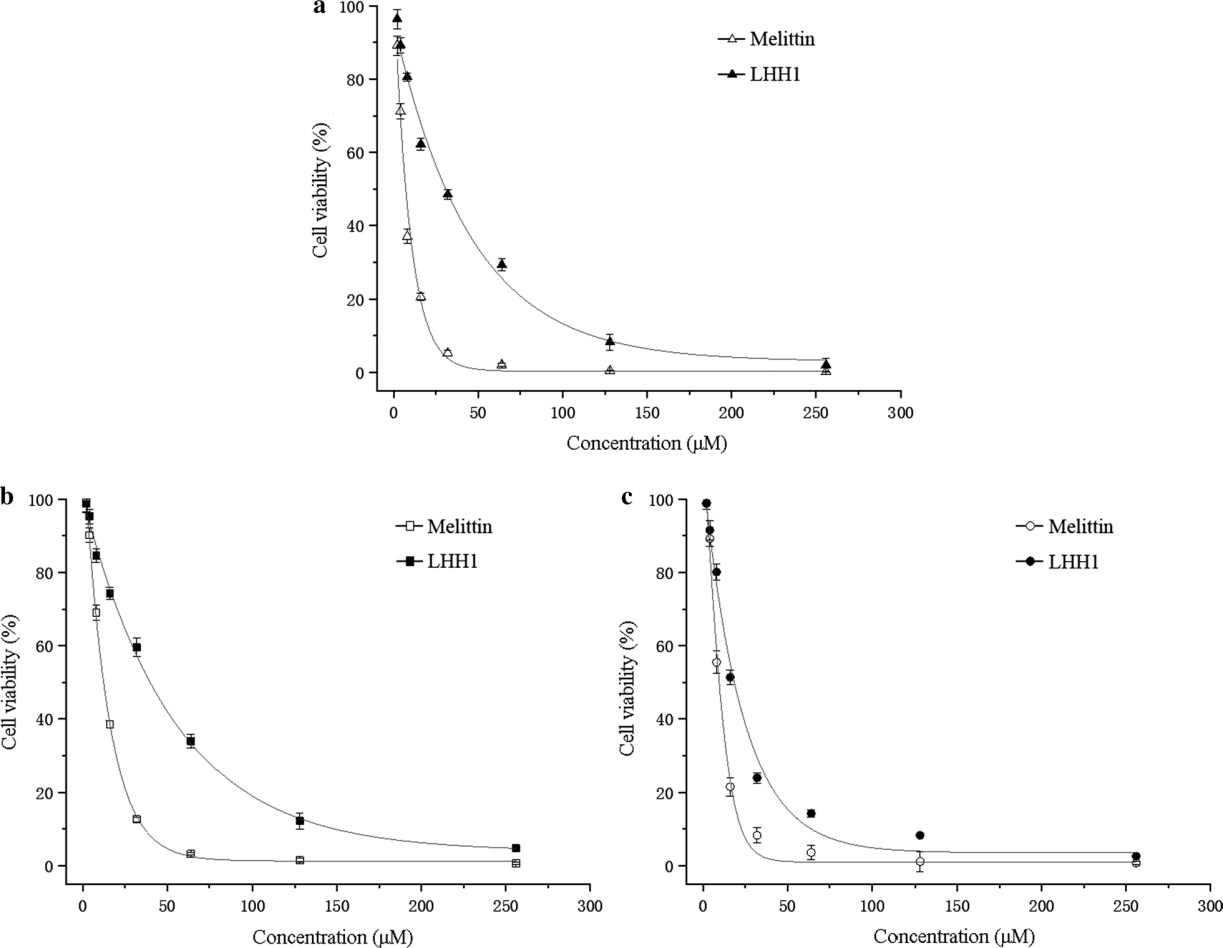
Effect of AMPs on cell viability of several cancer cells. MGC803 cells (**a**), HCT116 cells (**b**) and C666-1 cells (**c**) were treated with different concentrations of LHH1, respectively. Melittin was uused as a positive control

**Fig. 9:**
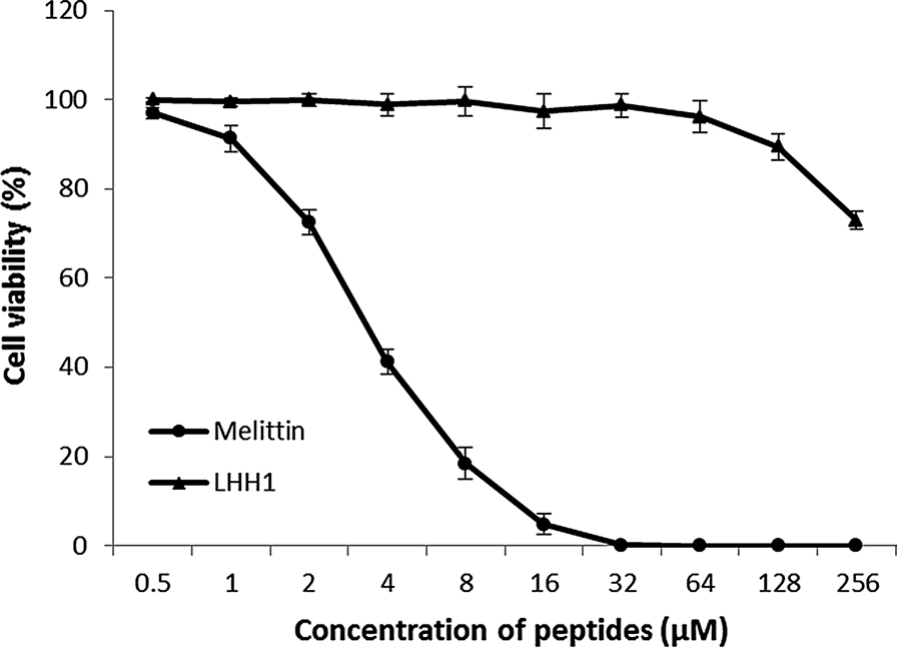
Toxicity of LHH1 to RAW264.7 cells. The human macrophage cell RAW264.7 was treated with a serial concentration of LHH1 or melittin

Anticancer cell activities of LHH1 were evaluated by MTT assays. Cell viability results were depicted in Fig. 8 and Table 5. LHH1 could dose-dependently inhibit the viabilities of C666-1, MGC803 and HCT116 cells. LHH1 exhibited the strongest inhibitory effect on C666-1 cells with an IC_50_ value of 18.27 μM, while IC_50_ values of inhibiting MGC803 and HCT116 cells were 31.55 μM and 40.83 μM, respectively. However, LHH1 exhibited weaker anticancer cell activities than that of melittin did. As positive control in this study, melittin could inhibit the growth of C666-1, MGC803 and HCT116 cells with IC50 values of 9.62 μM, 7.08 μM and 12.17 μM, respectively. Additionally, RAW 264.7 cells as a normal cell line were applied to detect the cytotoxic effect of LHH1 on normal cells in this study. As shown in Fig. 9, LHH1 had little influence on the viability of RAW 264.7 cells when the concentration of LHH1 was below 128 µM, which indicated that LHH1 was cell-selectively and concentration dependently cytotoxic.

**Table 5:** Anticancer cell activities of AMPs

Cancer cells	IC50 (µM)	
	LHH1	Melittin
HCT116	40.83	12.17
C666-1	18.27	9.62
MGC803	31.55	7.08

### Anticancer cell action of LHH1

**Fig. 10:**
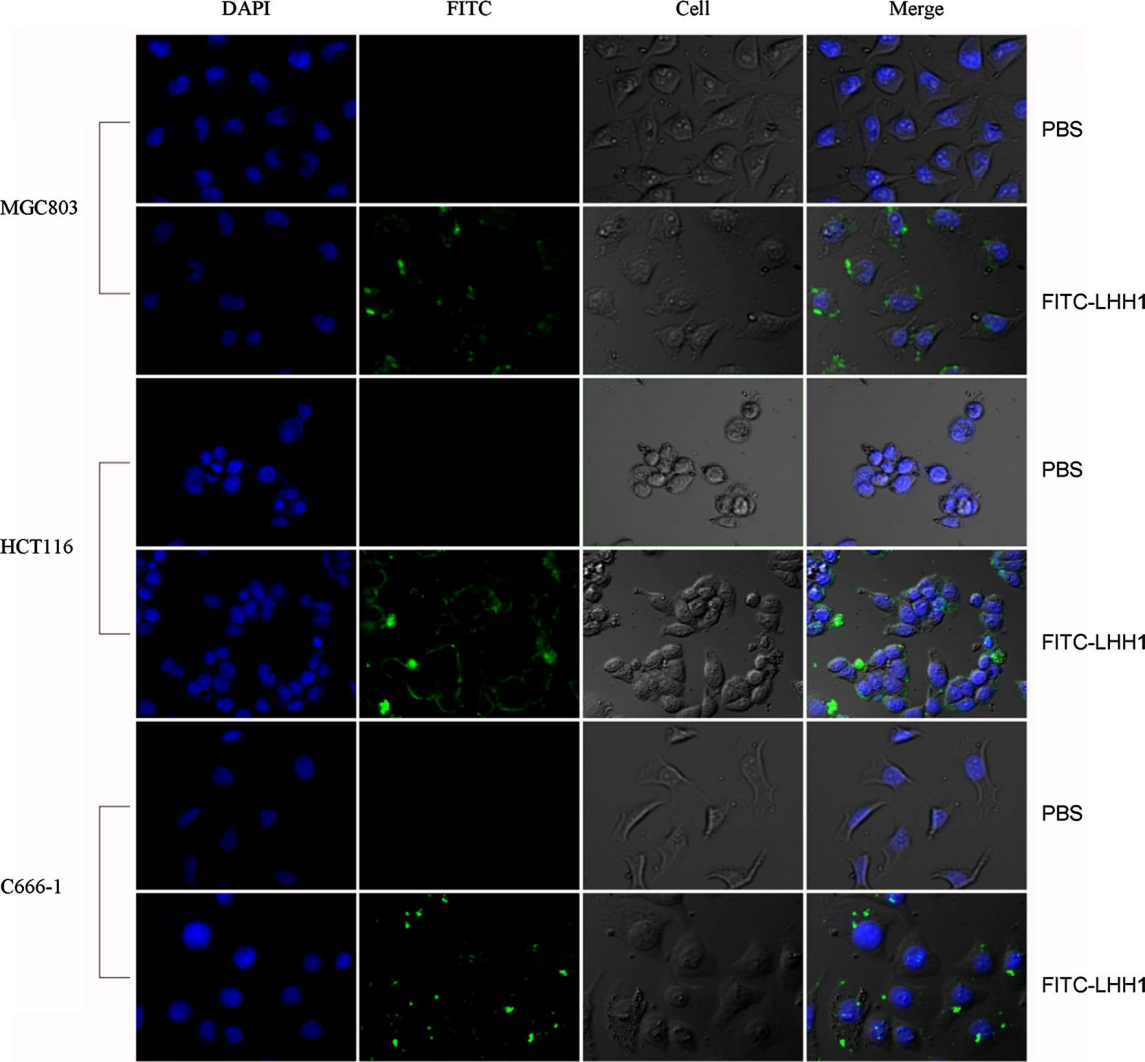
CLSM analysis of membrane destroying effects of LHH1 on cancer cells. The cancer cells were treated with FITC-labeled LHH1 or not and cell nucleus were stained with DAPI

To explore whether LHH1 had an influence on the membranes of cancer cells or not, three cancer cells were treated with FITC-labeled LHH1 and were observed with CLSM. As presented in Fig. 10, LHH1 could bind with the membranes of the three cancer cells, and the morphology of the cancer cells became blurred. Meanwhile, LHH1 could penetrate into the membranes of the three cancer cells.

**Fig. 11:**
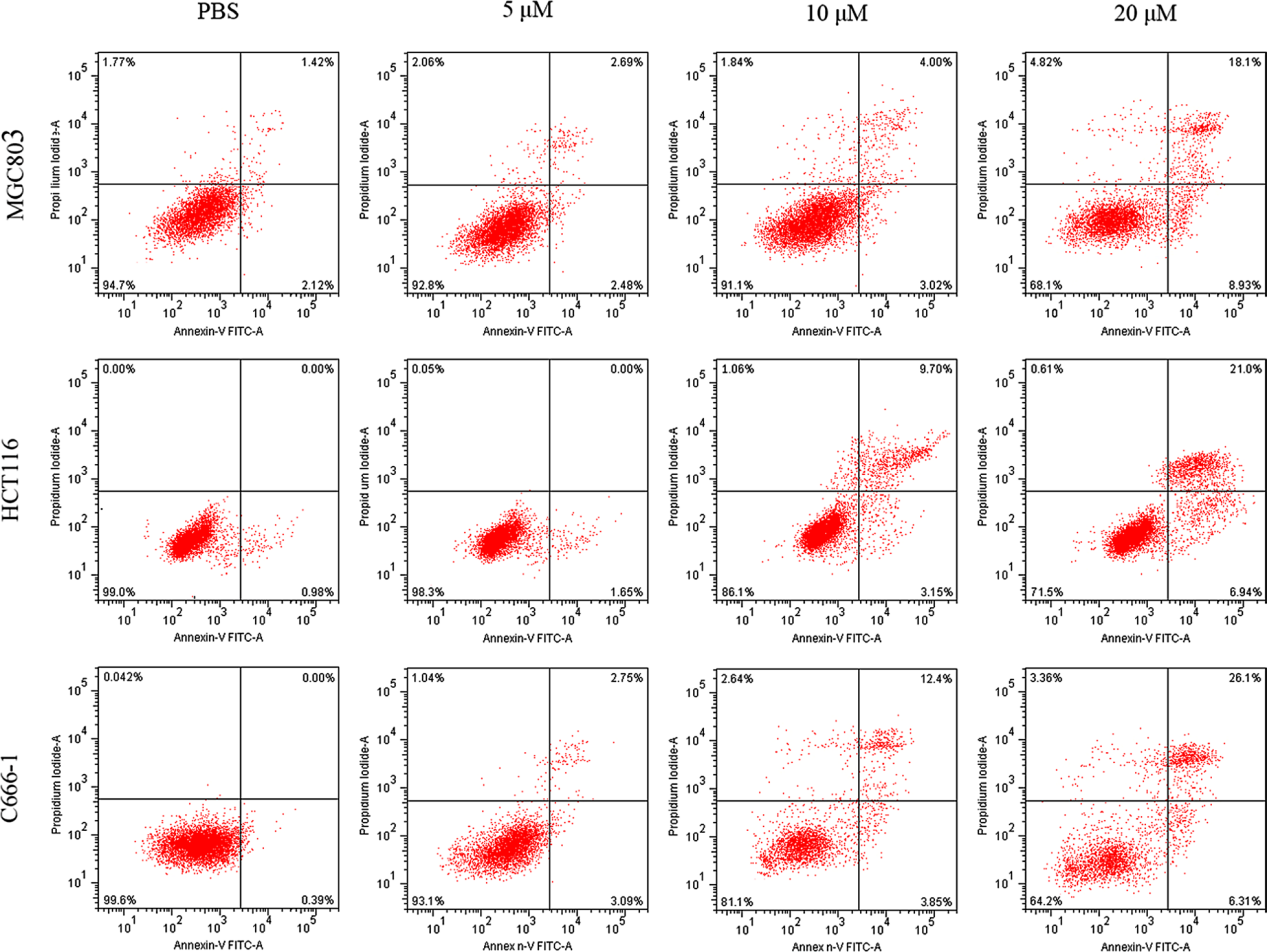
Flow cytometry analysis of apoptosis-promoting effect of LHH1 on cancer cells. The apoptosis rate of the three cancer cells treated with different concentrations of LHH1 (0 μM, 5 μM, 10 μM and 20 μM) was detected with flow cytometry using Annexin V-FITC/PI staining

To further clarify the mechanisms of inhibitory effects of LHH1, the three cancer cells were stained with Annexin V-FITC/PI and were analyzed by flow cytometry. As shown in Fig. 11, the viable MGC803 cells decreased from 92.8% to 68.1% with the increasing concentration of LHH1, while the ratio of LHH1-induced viable HCT116 cells was from 98.3% to 71.5%. The viable C666-1 cells decreased from 93.1% to 64.2% after the treatment of LHH1 at increasing concentrations. At 20 µM, LHH1 induced late apoptotic rates of 18.1%, 21.0% and 26.1% for MGC803, HCT116 and C666-1 cells, respectively. Moreover, small amounts of early apoptotic cells were observed for different cancer cells. Flow cytometry results displayed that LHH1-caused late apoptosis cells increased at a dose-dependent manner, which indicated that LHH1 exhibited anti-cancer cells activities partly via inducing late apoptosis of cancer cells.

## Discussion

AMPs have been recognized as the first line of defense against pathogens infection, and a growing body of evidence suggested that some AMPs showed anticancer and antiviral activities (Jiang et al. 2020). In the present study, LHH1 was a novel AMP that identified from the genome of *L. casei* HZ1 with potent antibacterial effect on *S. aureus* and anticancer potency on several cancer cells (Table 3 and Fig. 8), which may provide clear evidence for the application of LHH1 in certain foods as preservatives. Currently, there are 43 AMPs originated from *Lactobacillus* and 25 AMPs from *Lactococcus*, while only nisin and pediocin PA-1 are considered as potent bio-preservatives applied in food industry (Vijay Simha et al. 2012). The hemolytic and cytotoxic properties are two important criterias for evaluating the security of AMP as an additive agent or a drug. LHH1 in this study showed a low hemolytic activity and a low toxicity on RAW264.7 cells at its effective antibacterial activity (Table 3, Figs. 2, 9). Although the number of cells used in hemolysis test and antibacterial test is different, it can reflect the toxicity of antibacterial drugs to human red blood cells and pathogenic bacteria under pathogenic concentration of bacteria. Compared with high hemolytic activity of melittin, LHH1 showed a low hemolytic activity, and compared with high toxicity of melittin to normal cells, LHH1 possessed the characteristics of low toxicity to normal cells. Although melittin had better antibacterial activity than LHH1, it had much higher hemolytic activity than LHH1.

Many class II bacteriocins produced by LAB are synthesized as precursors containing a Gly-Gly/Ala motif leader sequence. The presence of a Gly-Gly/Ala motif containing peptide is closely related to the presence of a dedicated ATP-binding cassette (ABC) transporter (Dirix et al. 2004). Most of the precursor peptides belonging to the class II AMPs, contain a Gly-Gly/Ala motif leader sequence that are recognized and removed by their cognate ABC-transporter (van Belkum et al. 1997; Patton et al. 2008). The precursor of LHH1 contains 68 amino acid residues, while Gly-Gly motif is involved in its leader sequence. These characteristics are suitable for a protein expression by ABC-transporter from *L. casei* HZ1. In this study, LHH1 exhibited a higher antimicrobial capacity against *S. aureus* than those of other detected microbial strains did, and it displayed a low hemolysis at its effective anti-bacterial concentration (Table 3 and Fig. 2). Thus, the inhibitory action of LHH1 on *S. aureus* was further investigated.

**Fig. 12:**
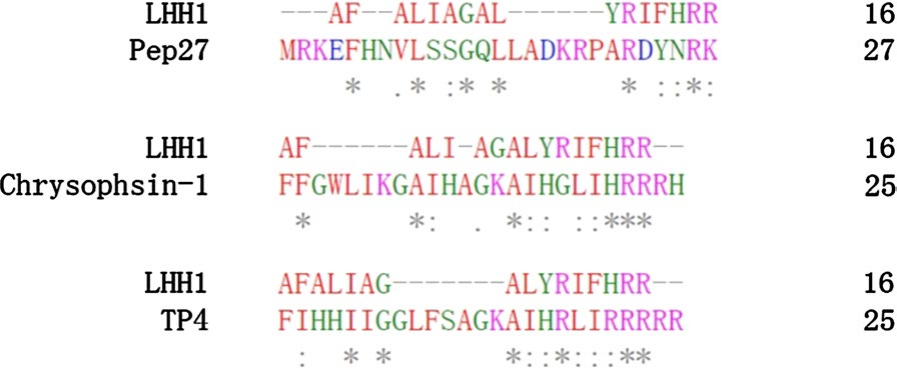
The sequence similarity alignment. The red ones are hydrophobic amino acids; The green ones are hydrophilic amino acid; * indicates highest similarity

The structural characteristics of AMPs play critical roles in their antimicrobial activity. Most of the bacteriocins found in LABs were defined as cationic polypeptides. The postively-charged LHH1 could specifically kill Gram-positive bacteria rather than Gram-negative bacteria, which may result from the differences in the composition of bacterial lipid membranes. On one hand, during the process of AMPs exerting their antibacterial activities, the cell wall outside the phospholipid membrane is the first hurdle that AMPs have to pass through. Before reaching the plasma membrane, most positively-charged AMPs have to penetrate into the outer membrane of gram-negative bacteria which contains lipopolysaccharides, or the cell walls of gram-positive bacteria containing acidic polysaccharides. On the other hand, the composition of phospholipids in the cell membrane of *S. aureus* and *E. coli* is very different, which is a major reason that results in different disturbing abilities of cationic AMPs to the cell membrane of *S. aureus* and *E. coli* (Chassaing and Cascales 2018). For example, the lipid membranes of *S. aureus* mainly consisted of negatively charged lipid such as cardiolipin (CL) and phosphatidylglycerol (POPG), whereas the lipid membranes of Gram-negative bacteria (ie. *E. coli*) were uncharged phosphatidylethanolamine (Epand 2008; Lohner and Prenner 1999). The selective bacterial cytotoxicity of cationic AMPs could be partly attributed to the net negative charge on the bacterial cell membranes, which facilitated the positive charges specifically target the negative-charged phospholipids on the membranes of Gram-positive bacteria (Arpornsuwan et al. 2014; Fields et al. 2018). Moreover, the increased net positive charge on the peptide might increase the binding affinity and stabilize the amphiphilic structure on the negative-charged lipid membrane (Ahn et al. 2006). A high proportion of positively charged amino acids at the C-terminus of LHH1 was speculated to play a pivotal role in the antimicrobial action for their cationic property. LHH1 has a similar residue of R-R/K with Pep27, Chrysophsin-1 and TP4 in the C-terminus of AMPs (Fig. 12). Arg residues was highlighted in previous researches for its critical role in the antimicrobial activity, which endow the peptides with cationic charges and hydrogen bonding properties necessary for interaction with the abundant anionic components of bacterial membrane (Chan et al. 2006). LHH1 possessed the capacity of binding with *S. aureus* and cancer (Figs. 5, 10). The present results were in accordance with previous researches that positively-charged amino acids in AMPs were required for binding to the negative-charged membrane through electrostatic interaction (Wimley 2010).

Hydrophobicity is a factor that plays pivotal roles in generating α-helix structure, and it is positively correlated with the membrane disrupting power of the peptides to a certain extent (Mattei et al. 2014; Ng and Deber 2014). In the study, 211 AMPs with anticancer activities, which were collected by APD3, were blasted with LHH1 to analyze the peptide sequence similarity. Three AMPs had the highest sequence similarity with LHH1, they were Pep27, Chrysophsin-1 and TP4 (Fig. 12). LHH1 has the highest rate of hydrophobic amino acid among the four AMPs. The hydrophobic amino acid rate of LHH1, Pep27, Chrysophsin-1 and TP4 are 62%, 29%, 48% and 44%. The sequence fragment of AFALIAGAL at the N-terminus of LHH1 contained a high proportion of hydrophobic amino acids. Generally, increased hydrophobicity of the non-polar face of an amphipathic α-helical peptide could improve antimicrobial activities (Dathe et al. 1997). As reported by Martina et al. hydrophobicity is positively correlated with the membrane disrupting power of the peptides to a certain extent (Bluhm 2015).

α-Helical conformation in AMPs is equally crucial in determining the antimicrobial activity of AMPs. Our present results showed that the α-helical content of LHH1 increased to above 85% when it was encountered with TFE and SDS micelle environment (Fig. 4 and Table 4), which indicated that LHH1 was an α-helix AMP. α-Helical structure might play a significant role in the antimicrobial effectiveness of LHH1 due to AMPs with α-helix could enhance the permeabilization of AMPs into the membrane of *S. aureus* (Park et al. 2004). The high content of α-helix structure will facilitate the formation of holes in the cell membrane of *S. aureus*, thus causing leakage of cytoplasm from the cell and inhibiting the growth of *S. aureus* (Park et al. 2004). Juba et al. reported that α-helix in AMPs could specifically interfere with the phospholipid fluidity, form a transient hole on the membrane and disrupt the membrane of *S. aureus*, thus inducing cytoplasmic membrane permeabilization and improving the antimicrobial activity (Juba et al. 1848). In this study, calcein leakage assay confirmed the interaction between LHH1 and the phospholipids on membrane of *S. aureus* (Fig. 3). In the present study, scanning electron microscope, confocal laser scanning microscope and flow cytometer were used to analyse the influence of LHH1 on *S. aureus*. The membrane of *S. aureus* was obviously disrupted after the treatment of LHH1 (Figs. 5, 6, 7), which provided distinct evidence that LHH1 could exhibit antimicrobial action by interacting with phospholipids, damaging the membrane of *S. aureus* and resulting in the leakage of cytoplasm. Furthermore, glycine in the sequence of LHH1 was positioned between hydrophobic amino acid and cationic amino acid fragments, which made the folding and distortion of secondary structure of LHH1 be available and was structurally beneficial for the inhibitory action of LHH1 against *S. aureus* (Rajeev Aurora 1994; Huang et al. 2014).

Antimicrobial peptides have been commonly considered to cause cancer cells to undergo rapid cell death through a direct cell membrane damaging effect. Variation in the composition of cell membrane has significant implications in the progression of cancer. Cancer cell membrane presented more negative charge and stronger membrane fluidity on cell membrane than normal cells. Thus, destroying the cell membrane of cancer cells could be an effective strategy to inhibit the growth of cancer cells. Our anti-cancer results confirmed that cationic LHH1 could specifically induce the apoptosis of several cancer cells including MGC803, HCT116 and C666-1 cells (Fig. 8). Moreover, LHH1 showed a low toxicity on RAW264.7 cells compared to melittin, the cell viability was above 95% at a concentration of 128 μM. However, the IC_50_ value of LHH1 against C666-1 cell was only 18.27 μM (Fig. 9), which indicated that LHH1 possessed a wider range of drug dosage than melittin. LHH1 exerted its anticancer activity partly by breaking the cell membranes (Fig. 10). The FCM result showed that Annexin-V could interact with the phosphatidylserine (PS), which indicated that the membrane of cancer cells was damaged by LHH1 (Fig. 11). As reported by Gaspar et al., anticancer activity of AMPs is mainly attributed to the electrostatic interactions between the peptides and the anionic membrane of cancer cells, and allow selective killing cancer cells (Gaspar et al. 2013).

In conclusion, LHH1 as a novel AMP that identified from the genome of *L. casei* HZ1, possessed a broad spectrum of antimicrobial and anticancer activities and its effective concentration was at low toxicity. Meanwhile, the present results indicated that the major antimicrobial and anticancer actions of LHH1 were via damaging cell membranes.

## Supplementary information

**Additional file 1: Figures S1–S10.** RP-HPLC and MS of the chemically synthesized peptides LHH1, LHH2, LHH3, LHH4 and FITC-LHH1, respectively. **Figure S11.** Schematic diagram of FITC-LHH1 fluorescein labeling.

## Data Availability

Please contact author for data requests.
