# Supplementary material for: LHH1, a novel antimicrobial peptide with anti-cancer cell activity identified from Lactobacillus casei HZ1
Source: AMB Express. 2020 Nov 11;10:204. doi: 10.1186/s13568-020-01139-8 (PMC7658291; doi:10.1186/s13568-020-01139-8)
Supplement: Supplementary file 1 — Additional file 1: Figures S1–S10. RP-HPLC and MS of the chemically synthesized peptides LHH1, LHH2, LHH3, LHH4 and FITC-LHH1, respectively. Figure S11. Schematic diagram of FITC-LHH1 fluorescein labeling. [file 13568_2020_1139_MOESM1_ESM.zip › Figure S10.pdf]

Product Name：FITC-LHH1　　　　　　　　　MW：2376.81

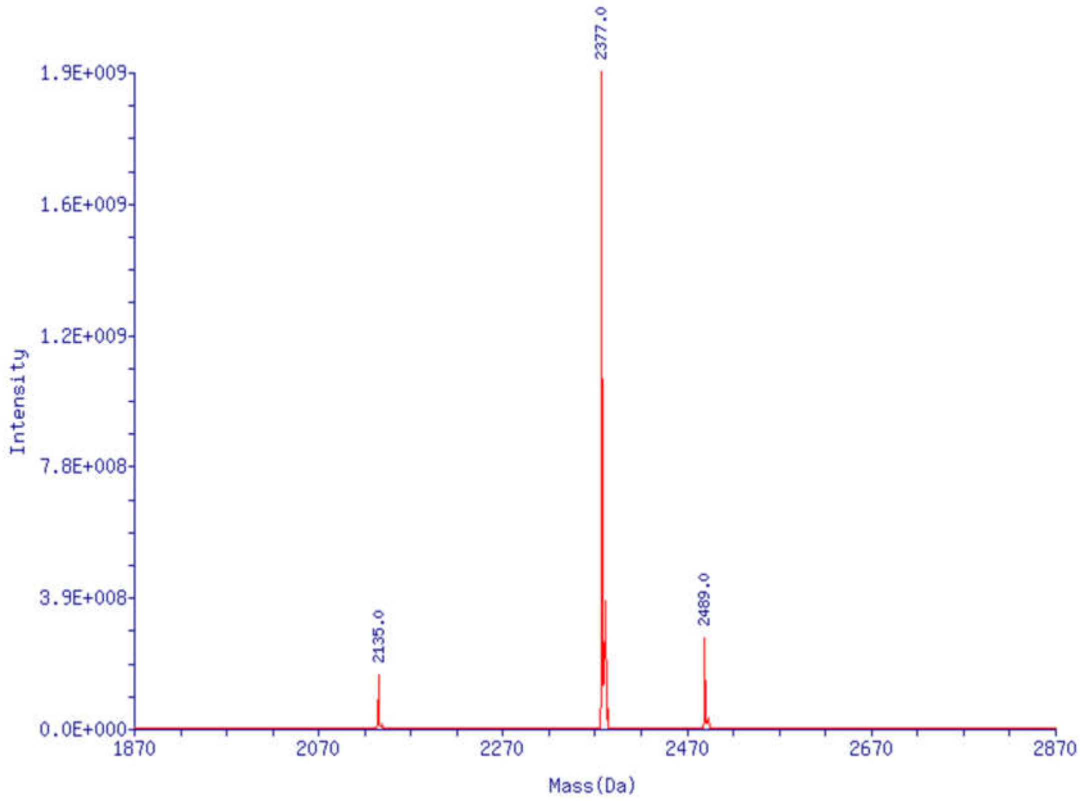


LCQ Deca XP MAX　　　ESI Source

Spray Voltage (kV): 5.02　　　Spray Current (μA):0.14　　　Sheath Gas Flow Rate: 35

Aux/Sweep Gas Flow Rate: 0　　　Capillary Voltage (V):14.85　　　Capillary Temp (°C): 250.00
