# Supplementary material for: LHH1, a novel antimicrobial peptide with anti-cancer cell activity identified from Lactobacillus casei HZ1
Source: AMB Express. 2020 Nov 11;10:204. doi: 10.1186/s13568-020-01139-8 (PMC7658291; doi:10.1186/s13568-020-01139-8)
Supplement: Supplementary file 1 — Additional file 1: Figures S1–S10. RP-HPLC and MS of the chemically synthesized peptides LHH1, LHH2, LHH3, LHH4 and FITC-LHH1, respectively. Figure S11. Schematic diagram of FITC-LHH1 fluorescein labeling. [file 13568_2020_1139_MOESM1_ESM.zip › Figure S3.pdf]

HPLC REPORT

Product Name	:	LHH2
Column	:	VYDAC-C18,4.6*250,5um
Solvent A	:	0.1%Trifluoroacetic in 100% Water
Solvent B	:	0.1%Trifluoroacetic in 100% Acetonitrile

Gradient :

	A	B
0.0min	80%	20%
20min	10%	90%
25min	0%	100%
30.0min	Stop	

Flow rate	:	1.0ml/min
Wavelength	:	220nm
Volume	:	20ul

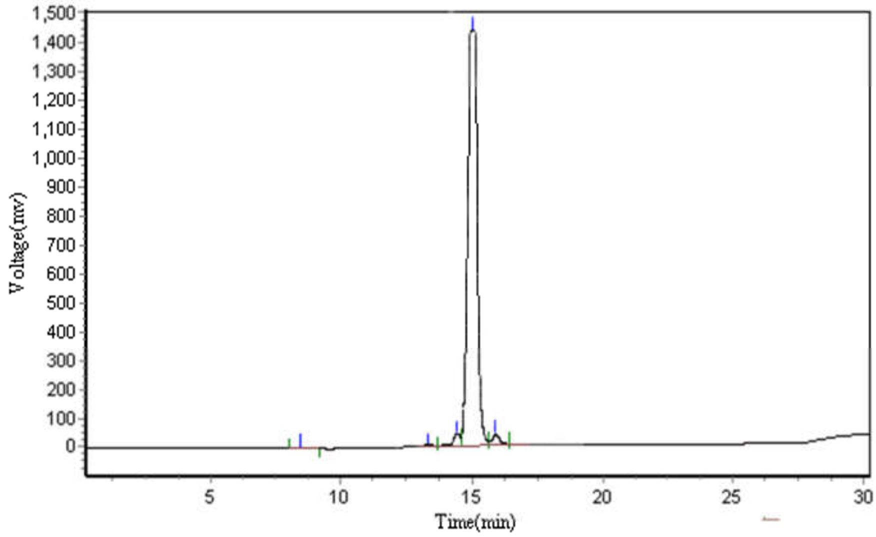

Results

Peak No.	Peak ID	Ret Time	Height	Area	Conc.
1		8.423	1127.764	21368.148	0.0551
2		13.380	5345.105	191592.891	0.4945
3		14.510	40983.762	737259.625	1.9027
4		15.092	1435209.250	36939320.000	95.3337
5		15.958	37689.605	857865.188	2.2140
**Total**			1520355.487	38747405.852	100.0000
