# Supplementary material for: LHH1, a novel antimicrobial peptide with anti-cancer cell activity identified from Lactobacillus casei HZ1
Source: AMB Express. 2020 Nov 11;10:204. doi: 10.1186/s13568-020-01139-8 (PMC7658291; doi:10.1186/s13568-020-01139-8)
Supplement: Supplementary file 1 — Additional file 1: Figures S1–S10. RP-HPLC and MS of the chemically synthesized peptides LHH1, LHH2, LHH3, LHH4 and FITC-LHH1, respectively. Figure S11. Schematic diagram of FITC-LHH1 fluorescein labeling. [file 13568_2020_1139_MOESM1_ESM.zip › Figure S5.pdf]

# HPLC REPORT

| | | |
|---|---|---|
| Product Name | : | LHH3 |
| Column | : | VYDAC-C18,4.6*250,5um |
| Solvent A | : | 0.1%Trifluoroacetic in 100% Water |
| Solvent B | : | 0.1%Trifluoroacetic in 100% Acetonitrile |
| Flow rate | : | 1.0ml/min |
| Wavelength | : | 220nm |
| Volume | : | 20ul |

Gradient :

| | A | B |
|---|---|---|
| 0.0min | 80% | 20% |
| 20min | 10% | 90% |
| 25min | 0% | 100% |
| 30.0min | Stop | |

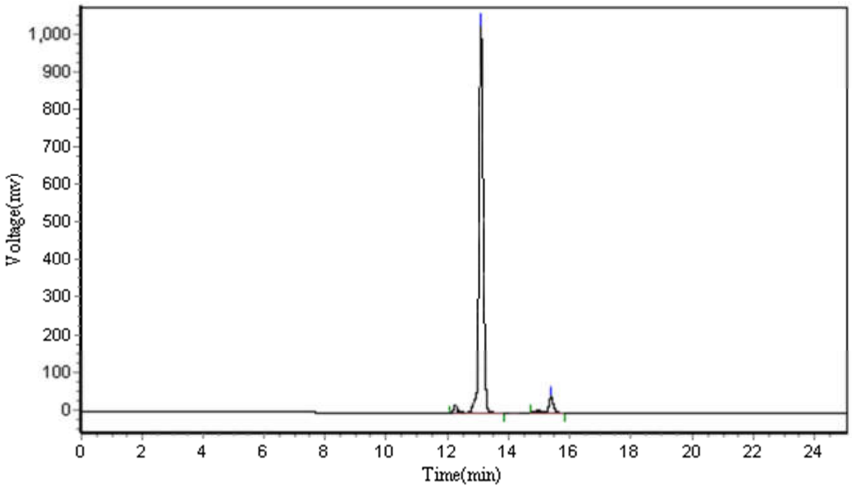


**Results**

| Peak No. | Peak ID | Ret Time | Height | Area | Conc. |
|---|---|---|---|---|---|
| 1 | | 13.068 | 1025443.625 | 10161958.000 | 95.6654 |
| 2 | | 15.868 | 42572.301 | 460443.313 | 4.3346 |
| Total | | | 1068015.926 | 10622401.313 | 100.0000 |
