# Supplementary material for: LHH1, a novel antimicrobial peptide with anti-cancer cell activity identified from Lactobacillus casei HZ1
Source: AMB Express. 2020 Nov 11;10:204. doi: 10.1186/s13568-020-01139-8 (PMC7658291; doi:10.1186/s13568-020-01139-8)
Supplement: Supplementary file 1 — Additional file 1: Figures S1–S10. RP-HPLC and MS of the chemically synthesized peptides LHH1, LHH2, LHH3, LHH4 and FITC-LHH1, respectively. Figure S11. Schematic diagram of FITC-LHH1 fluorescein labeling. [file 13568_2020_1139_MOESM1_ESM.zip › Figure S7.pdf]

HPLC REPORT

Product Name	LHH4		
Column	VYDAC-C18,4.6*250,5um		
Solvent A	0.1%Trifluoroacetic in 100% Water		
Solvent B	0.1%Trifluoroacetic in 100% Acetonitrile		
Gradient		A	B
	0.0min	80%	20%
	20min	10%	90%
	25min	0%	100%
	30.0min	Stop	
Flow rate	1.0ml/min		
Wavelength	220nm		
Volume	20ul		

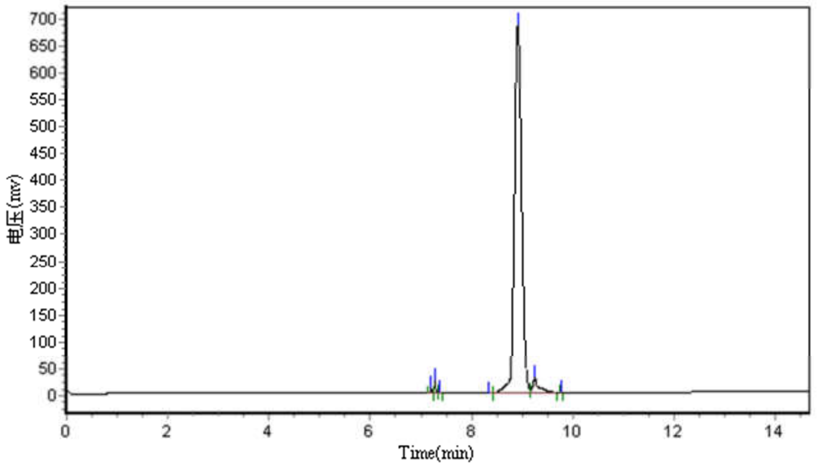

Results

Peak No.	Peak ID	Ret Time	Height	Area	Conc.
1		7.185	10722.061	19294.951	0.2722
2		7.258	24498.314	45775.535	0.6457
3		7.345	3562.401	6832.845	0.0964
4		8.327	114.508	413.552	0.0058
5		8.900	681915.688	6799788.000	95.9171
6		9.240	25926.012	217087.625	3.0622
7		9.773	20.348	42.700	0.0006
**Total**			746759.331	7089235.208	100.0000
