# Supplementary material for: LHH1, a novel antimicrobial peptide with anti-cancer cell activity identified from Lactobacillus casei HZ1
Source: AMB Express. 2020 Nov 11;10:204. doi: 10.1186/s13568-020-01139-8 (PMC7658291; doi:10.1186/s13568-020-01139-8)
Supplement: Supplementary file 1 — Additional file 1: Figures S1–S10. RP-HPLC and MS of the chemically synthesized peptides LHH1, LHH2, LHH3, LHH4 and FITC-LHH1, respectively. Figure S11. Schematic diagram of FITC-LHH1 fluorescein labeling. [file 13568_2020_1139_MOESM1_ESM.zip › Figure S9.pdf]

HPLC REPORT

Product Name	FITC-LHH1		
Column	VYDAC-C18,4.6*250,5um		
Solvent A	0.1%Trifluoroacetic in 100% Water		
Solvent B	0.1%Trifluoroacetic in 100% Acetonitrile		
Gradient		A	B
	0.0min	80%	20%
	20min	10%	90%
	25min	0%	100%
	30.0min	Stop	
Flow rate	1.0ml/min		
Wavelength	220nm		
Volume	20ul		

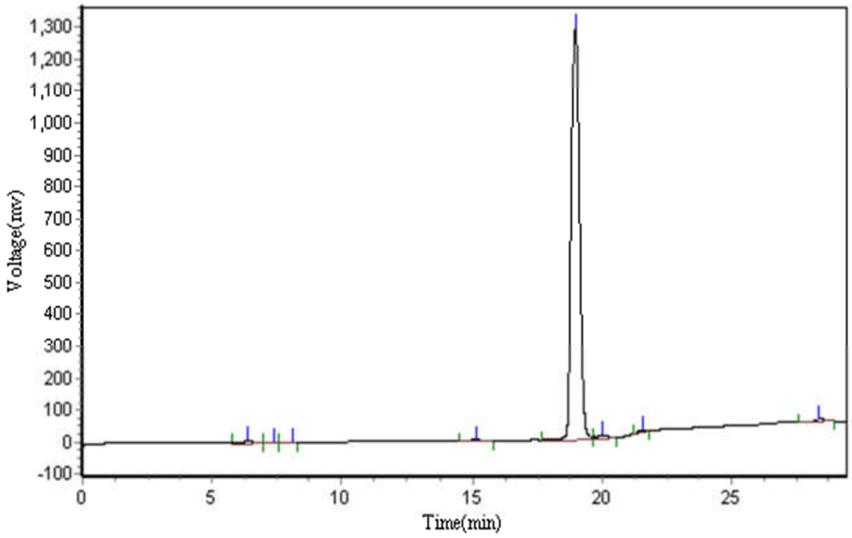

Results

Peak No.	Peak ID	Ret Time	Height	Area	Conc.
1		6.368	9350.480	370237.438	1.2397
2		7.405	2750.944	81565.188	0.2731
3		8.135	422.592	39044.652	0.1307
4		15.138	2492.332	45120.789	0.1511
5		18.967	1287522.250	28633928.000	95.8808
6		20.013	9994.888	277110.781	0.9279
7		21.593	6608.274	149459.016	0.5005
8		28.342	7509.394	267620.750	0.8961
Total			1326651.154	29864086.613	100.0000
